# EnzymeNet: residual neural networks model for Enzyme Commission number prediction

**DOI:** 10.1093/bioadv/vbad173

**Published:** 2023-11-24

**Authors:** Naoki Watanabe, Masaki Yamamoto, Masahiro Murata, Yuki Kuriya, Michihiro Araki

**Affiliations:** Artificial Intelligence Center for Health and Biomedical Research, National Institutes of Biomedical Innovation Health and Nutrition, Settu, Osaka 566-0002, Japan; Artificial Intelligence Center for Health and Biomedical Research, National Institutes of Biomedical Innovation Health and Nutrition, Settu, Osaka 566-0002, Japan; Graduate School of Science, Technology and Innovation, Kobe University, Kobe, Hyogo 657-8501, Japan; Artificial Intelligence Center for Health and Biomedical Research, National Institutes of Biomedical Innovation Health and Nutrition, Settu, Osaka 566-0002, Japan; Artificial Intelligence Center for Health and Biomedical Research, National Institutes of Biomedical Innovation Health and Nutrition, Settu, Osaka 566-0002, Japan; Graduate School of Science, Technology and Innovation, Kobe University, Kobe, Hyogo 657-8501, Japan; Graduate School of Medicine, Kyoto University, Kyoto, Kyoto 606-8501, Japan; Department of Cell Biology, National Cerebral and Cardiovascular Center, Suita, Osaka 564-8565, Japan

## Abstract

**Motivation:**

Enzymes are key targets to biosynthesize functional substances in metabolic engineering. Therefore, various machine learning models have been developed to predict Enzyme Commission (EC) numbers, one of the enzyme annotations. However, the previously reported models might predict the sequences with numerous consecutive identical amino acids, which are found within unannotated sequences, as enzymes.

**Results:**

Here, we propose EnzymeNet for prediction of complete EC numbers using residual neural networks. EnzymeNet can exclude the exceptional sequences described above. Several EnzymeNet models were built and optimized to explore the best conditions for removing such sequences. As a result, the models exhibited higher prediction accuracy with macro *F*_1_ score up to 0.850 than previously reported models. Moreover, even the enzyme sequences with low similarity to training data, which were difficult to predict using the reported models, could be predicted extensively using EnzymeNet models. The robustness of EnzymeNet models will lead to discover novel enzymes for biosynthesis of functional compounds using microorganisms.

**Availability and implementation:**

The source code of EnzymeNet models is freely available at https://github.com/nwatanbe/enzymenet.

## 1 Introduction

Enzymes are used with a wide range of industrial chemicals, pharmaceuticals, antibiotics, and food additives, and are essential mediators of metabolic pathways to biosynthesize functional substances using engineered microbes (Choi *et al.* 2015, Basso and Serban 2019). However, microbial metabolic pathways and enzymes are not necessarily optimal. Novel enzyme discovery is required to increase the production of target compounds (Otte and Hauer 2015, Ali *et al.* 2020). Moreover, the number of unannotated protein sequences is explosively increasing (Bateman *et al.* 2021). Therefore, a valid computational method to predict enzyme functions with high accuracy from sequence information is needed to help to discover novel enzymes within a huge number of unannotated sequences in the future.

Of these methods, one of the most basic approaches is machine learning which can learn various data and is suitable for mass predictions. Machine learning methods have been applied to predict various protein annotations (Almagro Armenteros *et al.* 2017, Kulmanov and Hoehndorf 2020). Then, several studies have been reported to predict Enzyme Commission (EC) numbers, one of the enzyme annotations (Dalkiran *et al.* 2018, Nursimulu *et al.* 2018, Ryu *et al.* 2019, Shi *et al.* 2023, Yu *et al.* 2023). EC numbers consist of four digits and are used to classify enzymes based on enzymatic reaction type. Yu *et al.* have recently proposed a contrastive learning based model, CLEAN, and the model can classify EC numbers and predict multiple functions for each sequence. Shi *et al.* have also developed a model, ECRECer, using multiple embedding representations extracted from protein sequences and a bidirectional gated recurrent unit neural network with an attention mechanism. The model can also predict non-enzymes in addition to the same features as CLEAN.

However, these studies have not discussed the evaluation of the sequences with numerous consecutive identical amino acids observed within unannotated sequences. The proteins with numerous consecutive identical amino acids might not have protein activity. Therefore, prediction models need to exclude the exceptional sequences from enzyme candidates for comprehensive enzyme annotation prediction. Without the operation, the sequences might be regarded as enzymes by prediction models and might remain in enzyme candidates in mass prediction of protein sequences. Moreover, the existing prediction models might not completely correctly predict more than a few thousand EC numbers, and a more valid model for EC number prediction is required to annotate enzyme features for a vast number of unannotated protein sequences.

Here, EnzymeNet models using residual neural networks (ResNet) were developed to predict EC numbers while removing proteins except for enzymes from sequence candidates used in enzymatic reaction prediction (He *et al.* 2016). ResNet which includes multiple convolutional neural network (CNN) layers has been demonstrated in protein structure and ligand-binding site predictions (Hanson *et al.* 2019, Shi *et al.* 2020) and can address vanishing gradient problem occurring in deep learning models with deeper layers. Moreover, several CNN models built from the image-like features which were transformed to one-hot encoding from sequence information have been demonstrated in various enzyme annotation predictions (Ryu *et al.* 2019, Kulmanov and Hoehndorf 2020). Enzyme sequence information might consist of structural information because several reports enabled to predict protein structures from sequence information using deep learning (Baek *et al.* 2021, Tunyasuvunakool *et al.* 2021). Therefore, EnzymeNet models were built using ResNet which can learn sequence data while capturing extensive enzyme features.

**Figure 1.**
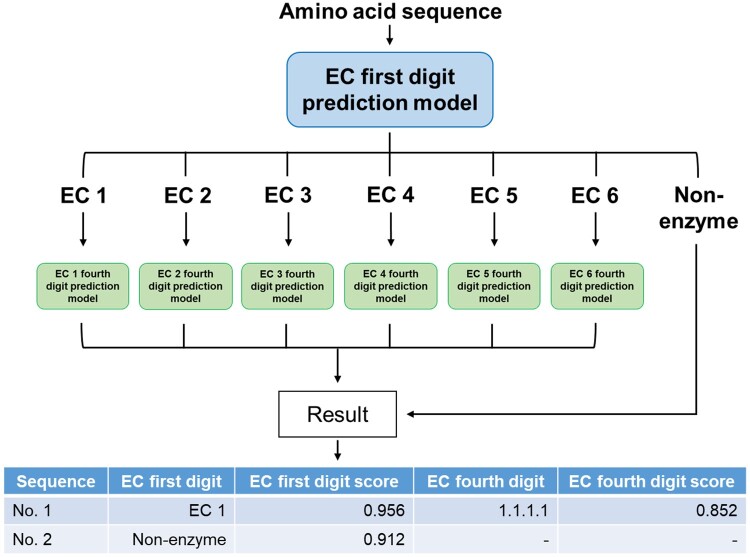
Scheme of two-step EC number prediction using EnzymeNet.

The EnzymeNet models predict EC numbers in two steps: (i) EC number first digit or negative and (ii) complete EC number prediction (Fig. 1). Moreover, the models exclude the exceptional sequences with numerous consecutive identical amino acids in the first step. Therefore, the optimized condition of EnzymeNet models to remove such sequences was determined using several different negative datasets. The models were more accurate for extensive enzyme sequences with lower similarity to our training data than four previously reported models based on machine learning and sequence similarity methods (Dalkiran *et al.* 2018, Nursimulu *et al.* 2018, Ryu *et al.* 2019, Sanderson et al. 2023). The experimental evaluation of enzyme candidates predicted by EnzymeNet in the future can help to discover novel enzymes.

## 2 Methods

### 2.1 Data collection

#### 2.1.1 Data for prediction of EC number first digits and negative sequences

To build positive data, 5 610 630 enzyme sequences for each EC class were collected from Kyoto Encyclopedia of Genes and Genomes (KEGG) GENES (Kanehisa and Goto 2000) released on July 2019 by KEGG FTP Academic Subscription. KEGG data are freely available for academic users. The enzyme sequences are also registered in the other databases (Supplementary Fig. S1) and the part of the sequences have been annotated by KEGG (Kanehisa *et al.* 2002). There are seven first digit EC number classes referred to as EC 1 to EC 7. EC 7 enzymes were not included in any of the data because too few enzymes were registered in KEGG. Enzyme sequences that were duplicated, with multiple EC numbers, or included non-canonical amino acids were removed and the length of amino acid residues is limited from 100 to 1000.

To keep data balanced, highly similar enzyme sequences were omitted by clustering at 90% identity using CD-HIT (Li and Godzik 2006) and then only a single enzyme sequence from each cluster was included. More than 80% of the EC numbers consisted of fewer than 800 sequences. Therefore, similar sequences were removed by decreasing the identity until the number of sequences within each EC number was fewer than 800. As a result, 1 049 807 unique enzyme sequences were used to build and evaluate EnzymeNet models.

To remove non-enzyme protein sequences and the exceptional sequences in the first prediction of EnzymeNet, negative data were built in three ways as follows: (i) Non-enzyme, (ii) random substitution, and (iii) consecutive substitution. Three random substitution and three consecutive substitution datasets were built to optimize the models for the first prediction.

##### 2.1.1.1 Non-enzyme

Proteins except for enzyme sequences which are freely available were collected from Swiss-Prot released in 2021 (Bateman *et al.* 2021). The sequences that were duplicated or included non-canonical amino acids were removed and the length of amino acid residues was limited from 100 to 1000. Only a single enzyme sequence from each cluster was used after clustering at 90% identity to remove the sequence redundancy in the data. As a result, 142 378 non-enzyme sequences were used.

##### 2.1.1.2 Random substitution

About 16 964 sequences were randomly extracted from the enzyme sequences included in the positive data. For each sequence, 20% of the random amino acids of the sequence were substituted with the other amino acids (Supplementary Fig. S2a). The position and type of the substituted amino acids were randomly selected. This strategy was inspired by masked language models, such as Bidirectional Encoder Representations from Transformers (BERT). The BERT is pretrained by randomly masking some of the tokens from input data, and the objective of the training is to predict the original vocabulary of the masked word based only on its context (Devlin *et al.* 2019). Therefore, to make EnzymeNet models understand original amino acid patterns of enzymes and the other sequence patterns, the artificial random substitution sequences were built. Moreover, 10% and 40% random substitution datasets were generated to evaluate the effect of the rate of substituted amino acids on this prediction.

##### 2.1.1.3 Consecutive substitution

About 16 964 sequences were randomly extracted from positive data. For each sequence, 50%∼80% of the amino acids in the sequence were substituted with consecutive identical amino acids (Supplementary Fig. S2b). The position, type, and rate of the substituted amino acids were randomly selected. Previously reported models were not evaluated using such sequences, which are found within unannotated sequences. Therefore, the current models enabled to remove the sequences. Moreover, 1%∼25% and 26%∼49% consecutive substitution datasets were generated to explore the relationship between prediction accuracy and the rate of substituted amino acids.

All positive and negative data were merged. All data were randomly split into training, validation, and test data at an approximate ratio of 8:1:1 (Table 1). Training, validation, and test data were used for building models, evaluating all models in training and evaluating all models after training, respectively. Most of the enzyme sequences in positive data are also registered in National Center for Biotechnology Information and UniProt (Supplementary Fig. S1). Common test data consisted of the test data of positive data and non-enzymes for prediction of EC number first digits, and the data for the same number of artificial negative test data extracted from each condition of building artificial negative data (Supplementary Table S1). The common test data were used to evaluate six EnzymeNet models and to determine the optimal models and to compare the models to previously reported models in the first prediction.

**Table 1.** Datasets size of EnzymeNet.

Step	Type	EC	Training	Validation	Test
First prediction		EC 1	180 177	22 160	22 161
		EC 2	279 647	34 487	34 473
	Positive	EC 3	206 177	25 408	25 418
		EC 4	81 624	10 057	10 054
		EC 5	50 103	6180	6182
		EC 6	44 521	5489	5489
		Non-enzyme	113 881	14 225	14 272
	Negative	Random substitution	13 585	1695	1684
		Consecutive substitution	13 580	1710	1674
Second prediction		EC 1 fourth digit	122 858	14 961	14 961
		EC 2 fourth digit	284 539	35 139	35 139
	Positive	EC 3 fourth digit	222 466	27 511	27 511
		EC 4 fourth digit	658,52	8065	8065
		EC 5 fourth digit	55 187	6825	6825
		EC 6 fourth digit	107 511	13 380	13 380

#### 2.1.2 Data for prediction of complete EC numbers

Positive data for EC number first digit prediction were separated by each EC number fourth digit. Highly similar enzyme sequences were omitted by clustering at 90% identity to decrease sequence redundancy. Moreover, the sequences with EC numbers that contained much fewer sequences in each EC number fourth digit were removed. The data were randomly split into training, validation, and test data at an approximate ratio of 8:1:1 (Table 1).

### 2.2 Model construction

**Figure 2.**
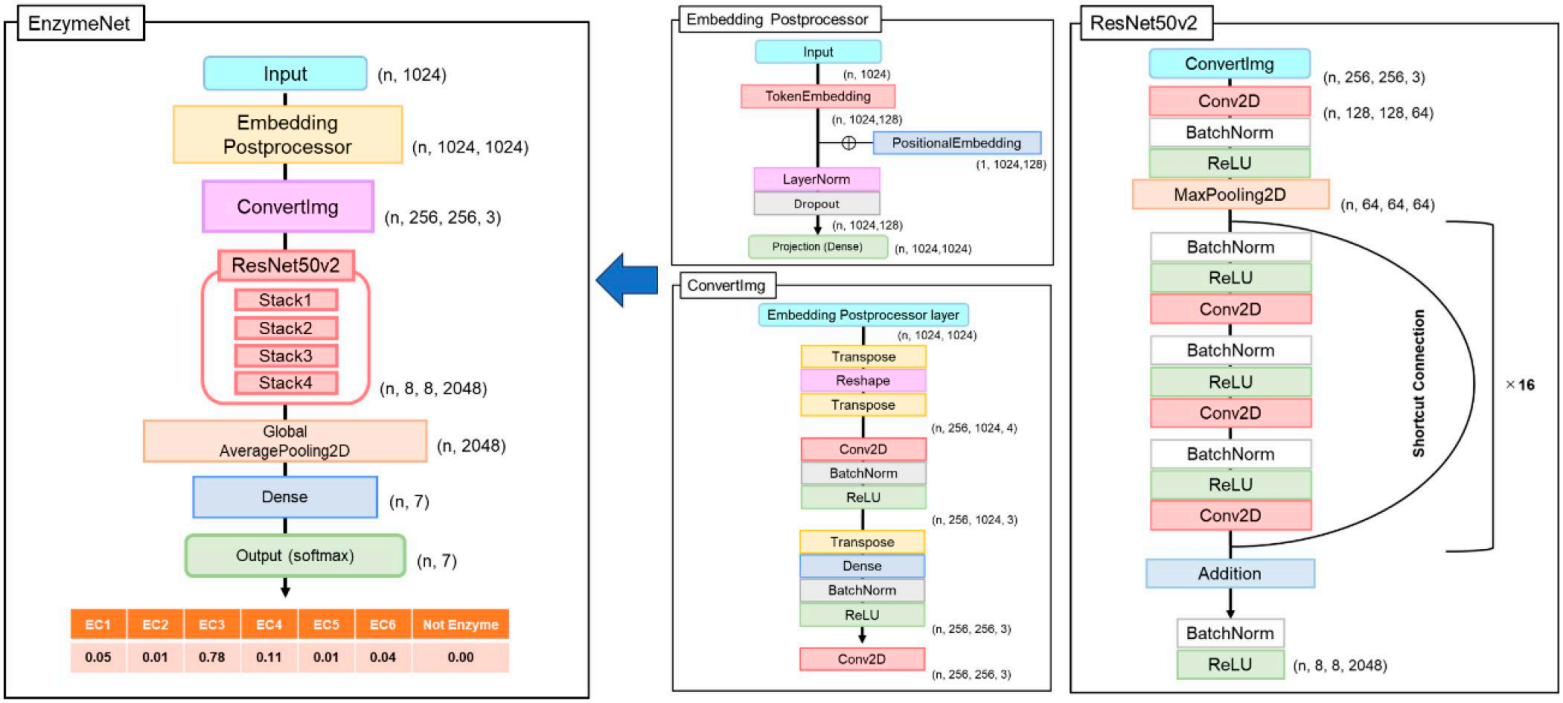
EnzymeNet structure in the first prediction using ResNet50v2.

EnzymeNet models which were built using ResNet50v2 (He *et al.* 2016) consisted of two predictions: (i) prediction of EC number first digits and negative and (ii) prediction of complete EC numbers. The model structure in the first prediction is shown in Fig. 2. In Embedding Postprocessor layer (Lan *et al.* 2020), each amino acid included in each sequence was transformed into tokens which could be treated by deep learning. Zero padding was used for the sequences with less than 1024 residues. The tokens were transformed to (*n*, 1024, 128) feature maps. The positional information of each amino acid which is important for protein activity was added to the feature maps by Positional Embedding, and (*n*, 1024, 1024) feature maps were outputted. Next, in ConvertImg layer, feature maps were transformed to image-like (*n*, 256, 256, 3) feature maps, which were passed through ResNet50v2. Several studies have reported various biological predictions using CNN which has been often used in image recognition (Ryu *et al.* 2019, Kulmanov and Hoehndorf 2020). ResNet can address vanishing gradient problem occurring in deep learning models with deeper CNN layers. Therefore, ResNet which was an expanded CNN model was used to build EC number prediction models. From the final layer, the scores for seven classes were then outputted. Moreover, six models referred to as EnzymeNet version 01 to 06 (v_01 to v_06) models were built from the same positive and non-enzyme datasets, and different artificial datasets obtained under different conditions of random and consecutive substitutions to explore the optimal condition in the first prediction (Table 2).

**Table 2.** Type of artificial negative datasets used in six EnzymeNet versions in the first prediction.

Model	Random substitution	Consecutive substitution
EnzymeNet version_01 (v_01)	N/A	N/A
EnzymeNet version_02 (v_02)	10%	50% ∼ 80%
EnzymeNet version_03 (v_03)	20%	1% ∼ 25%
EnzymeNet version_04 (v_04)	20%	26% ∼ 49%
EnzymeNet version_05 (v_05)	20%	50% ∼ 80%
EnzymeNet version_06 (v_06)	40%	50% ∼ 80%

The EnzymeNet models in the second prediction were built applying transfer learning for the first step’s EnzymeNet models which predicted with higher accuracy in the common test evaluation. For each model, six models for EC 1 to EC 6 were built. When EnzymeNet models predict EC numbers for a sequence, EC number first digit is predicted by the first prediction model, and then complete EC number is predicted by one of the six models selected from the first results (Fig. 1). If a result of the first prediction is negative, the second prediction is not performed. The all models in this study were built using TensorFlow (Abadi *et al.* 2016). A categorical cross-entropy loss function was used to train the models, and trainable parameters were updated for each batch.

### 2.3 Model evaluation

EnzymeNet models were evaluated in three ways as follows. First, EnzymeNet v_01 to v_06 models for EC number first digit and negative predictions were evaluated using test data. All models were also evaluated using common test data to determine the optimized models for these predictions. Second, for only complete EC number prediction, the EnzymeNet models were evaluated using the test data for the second prediction. The EC 1 to EC 6 models of each EnzymeNet model were evaluated using each EC fourth digit dataset in the complete EC number prediction, respectively. The evaluation was not used for evaluating the EnzymeNet models to the other EC number prediction models in the complete prediction. Then, the EnzymeNet models combining the first and the second prediction models were evaluated to confirm the ability for both predictions by Continuous Test, in which the models firstly predicted EC number first digits or negative using the second prediction’s test data, and predicted complete EC numbers using only correctly predicted test data in the first prediction. The incorrect test samples in the first prediction were not predicted in the next prediction. Accuracy, *F*_1_ score, Precision, Recall, and Matthews correlation coefficient (MCC) were used for the evaluations. The detailed information of evaluation parameters is shown in Supplementary Data.

Moreover, EnzymeNet models were compared with four EC number prediction models, DeepEC (Ryu *et al.* 2019), DETECT v2 (Nursimulu *et al.* 2018), ECPred (Dalkiran *et al.* 2018), and ProteInfer (Sanderson et al. 2023) using same test datasets. All models were evaluated using three ways. First, for simple EC number prediction, the common test data for EC number first digit prediction was used in the evaluation of EC number first digit and negative predictions, while the test data for complete EC number prediction was used in the evaluation of complete EC number prediction. In the complete prediction, the EnzymeNet models were evaluated in the same way as the Continuous Test because of the two-step prediction. The other models which had already been trained in each report were used.

DeepEC, ECPred, and Proteinfer were based on machine learning methods while DETECT v2 was based on sequence similarity strategy (Camacho *et al.* 2009). DETECT v2 and ProteInfer could not predict negative samples and therefore, the test samples whose scores were not outputted by these models were regarded as negative. The test samples whose scores were not outputted by DeepEC and ECPred were regarded as incorrect because these models could predict negative samples. Moreover, EnzymeNet models were compared to two ensemble methods combined with four previous models in only complete EC number prediction. The first ensemble method (Ensemble 1) was evaluated using a majority rule. If the four models predicted EC 1.1.1.1, EC 1.1.1.2, EC 1.1.1.3, and EC 1.1.1.2 for a test sample, respectively, the prediction result of the first ensemble method was EC 1.1.1.2. If the two models predicted one EC number (EC 1.1.1.1 and EC 1.1.1.1) and the other models predicted another EC number (EC 1.1.1.2 and EC 1.1.1.2) for a test sample, or if all models predicted different EC numbers, the result was randomly selected. In the second ensemble method (Ensemble 2), the test samples correctly predicted by at least one of the four models were regarded as correct, while the other cases were regarded as incorrect. The performances of all models were evaluated using Accuracy, Macro *F*_1_ score, Macro Precision, and Macro Recall. To compare the accuracy for prediction of all EC numbers in test data to all models, these values for all EC numbers were calculated using the number of EC numbers.

Second, to evaluate these models for only specific EC number prediction, these models were evaluated using two test data, which the enzyme sequences with high similarity to the training data are removed from, by lowering the sequence identity threshold using CD-HIT. One data was the enzyme and non-enzyme sequences extracted from common test data for prediction of EC number first digits and the other data was test data for prediction of complete EC numbers. Finally, since all EC numbers included in our test datasets could not be necessarily predicted by the previously reported models, all models in addition to EnzymeNet models were also compared the results of the only predictable EC numbers which were outputted from the test datasets using each model.

## 3 Results

### 3.1 EC number first digit and negative predictions using EnzymeNet models

Supplementary Fig. S3 shows loss function curves for training and validation in the first prediction. The validation loss function decreased as epochs proceed. The results indicated that all EnzymeNet models for the prediction do not overfit. Test results are shown in Supplementary Table S2. The model performances of all versions increased as epochs proceeded. The models were built using 1500, 1300, 1400, 1500, 1400 and 1500 epochs, respectively, where the MCCs were highest and the other values were relatively higher. Prediction accuracies showed no significant differences among the models.

Next, the models were evaluated using common test data (Table 3 and Supplementary Table S3). The results of the overall first step prediction maintained constant high accuracy among EnzymeNet models, while the prediction results of the negative samples varied. EnzymeNet v_03 model was more accurate for negative sequences than the other models. EnzymeNet v_01 model which learned only non-enzyme dataset as negative data predicted artificial negative samples with much lower accuracy. The EnzymeNet v_03 and v_05 models were regarded as optimized models in the first step prediction because the models more correctly predicted both all test sequences and artificial sequences. Supplementary Table S4 shows the common test results using the two models for each class. EnzymeNet v_06 model was not selected as an optimized model because the model learned the more different artificial sequences from original enzyme sequences and more easily classified the sequences than the other models. All models predicted consecutive substitution samples with higher accuracy than random substitution samples.

**Table 3.** Common test results of the first step using six EnzymeNet models.

Model	Epoch	Macro *F*_1_ score	Macro Precision	Macro Recall	MCC
EnzymeNet v_01	1500	0.885	0.885	0.884	0.849
EnzymeNet v_02	1300	0.869	0.855	0.883	0.832
EnzymeNet v_03	1400	0.885	0.891	0.878	0.852
EnzymeNet v_04	1500	0.868	0.855	0.882	0.841
EnzymeNet v_05	1400	0.883	0.886	0.881	0.851
EnzymeNet v_06	1500	0.889	0.894	0.884	0.854

Model	Accuracy of random substitution samples	Accuracy of consecutive substitution samples	Accuracy of all artificial negative samples
EnzymeNet v_01	0.090	0.561	0.325
EnzymeNet v_02	0.443	0.829	0.636
EnzymeNet v_03	0.445	0.910	0.678
EnzymeNet v_04	0.250	0.746	0.498
EnzymeNet v_05	0.383	0.777	0.580
EnzymeNet v_06	0.398	0.784	0.591

### 3.2 Complete EC number prediction using EnzymeNet models

Supplementary Fig. S4 shows loss function curves for training and validation in the second prediction using EnzymeNet v_05 models. The results of EnzymeNet v_03 models were similar to that of EnzymeNet v_05 models. Unlike the first prediction, the validation loss functions of s models for EC 1 to EC 6 insufficiently decreased in comparison to the training loss. However, all models were not regarded as overfitting, because all validation loss functions did not significantly increase. The EnzymeNet v_05 models for EC 1 to EC 6 were built using 400, 500, 400, 400, 90, and 300 epochs, respectively (Supplementary Table S5). On the other hand, the EnzymeNet v_03 models were built using 500, 500, 400, 350, 90, and 450 epochs, respectively. Both models also predicted test data with high accuracy in the second prediction although the accuracies were lower than that of EC number first digit prediction.

**Figure 3.**
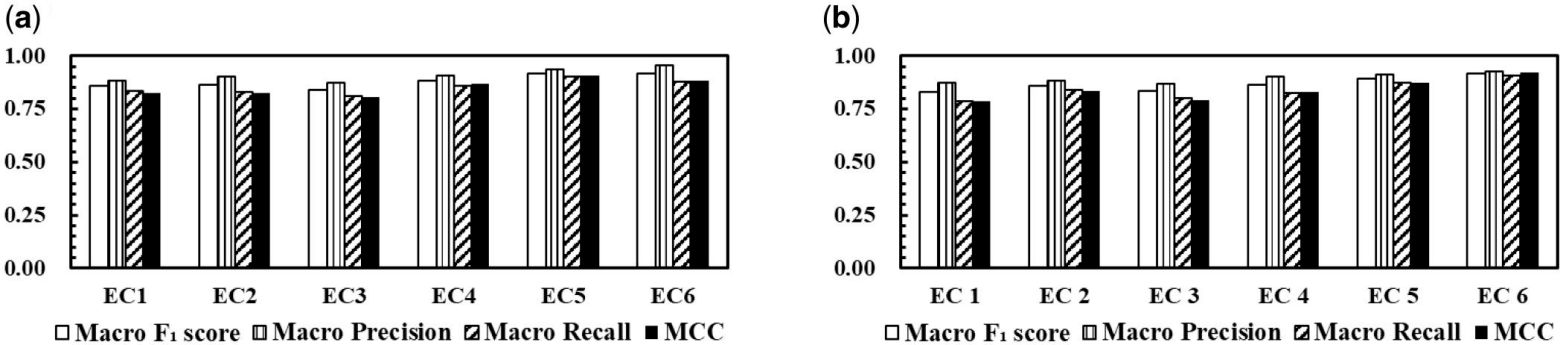
Continuous Test results of (a) EnzymeNet v_03 models and (b) EnzymeNet v_05 models. In the Continuous Test, the models firstly predicted EC number first digits or negative using the second prediction’s test data and predicted complete EC numbers using only correctly predicted test data in the first prediction.

Continuous Test results of EC number first digits and complete EC numbers for the models are shown in Fig. 3. The data of complete EC number prediction was used in this evaluation. The incorrect test samples in the first predictions were not performed in the next predictions. As a result, the prediction accuracies were slightly lower than in only complete EC number prediction but remained high. As with the first prediction, EnzymeNet v_03 models were more accurate than EnzymeNet v_05 models.

### 3.3 Comparative evaluation of EC number prediction

**Figure 4.**
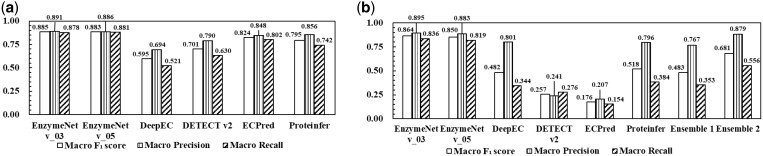
Comparative results of (a) EC number first digit and negative predictions and (b) complete EC number prediction.

As a benchmark, the EnzymeNet models were compared with DeepEC, DETECT v2, ECPred, and ProteInfer using common test data, and test data for prediction of complete EC numbers. Figure 4a and Supplementary Fig. S5 and Table S6 show the comparative results of common test data. Both EnzymeNet models exhibited higher test prediction accuracy and higher both Macro Precision and Macro Recall. The accuracies of DeepEC and DETECT v2 were lower than those of other models and the Macro Recalls were lower than the Macro Precisions. Moreover, the ability to classify non-enzyme and random substitution sequences using EnzymeNet models was lower than that of DETECT v2 and ProteInfer (Supplementary Fig. S5 and Table S6). Random substitution sequences tended to be more incorrectly predicted than consecutive substitution sequences. Both EnzymeNet models predicted correctly more consecutive substitution sequences than the other models.

Next, the prediction results of complete EC number prediction are shown in Fig. 4b. EnzymeNet models were also compared to two ensemble methods as described in Section 2.3. Both EnzymeNet models showed higher prediction accuracy with Macro *F*_1_ scores up to 0.850 than the other models and ensemble methods. The conditions of negative artificial datasets in EnzymeNet v03 models were more suitable for EC number prediction than EnzymeNet v_05 model because of the higher accuracies of all evaluations. On the other hand, the accuracies of the other models in the second prediction decreased much more than those of the first prediction. The test enzyme sequences included 2591 EC numbers and some EC numbers were easy to predict using the previously reported models. DETECT v2 predicted 468 EC numbers with higher accuracy than EnzymeNet models and the other models. For example, the reactions involved in polymer, protein, RNA, and DNA, which EnzymeNet models predicted with low accuracy, were also included. However, the *F*_1_ scores of 1953 of 2591 EC numbers for EnzymeNet v_03 or v_05 models were higher. All EC number results of the benchmark evaluation are shown in Supplementary Sheet.

**Figure 5.**
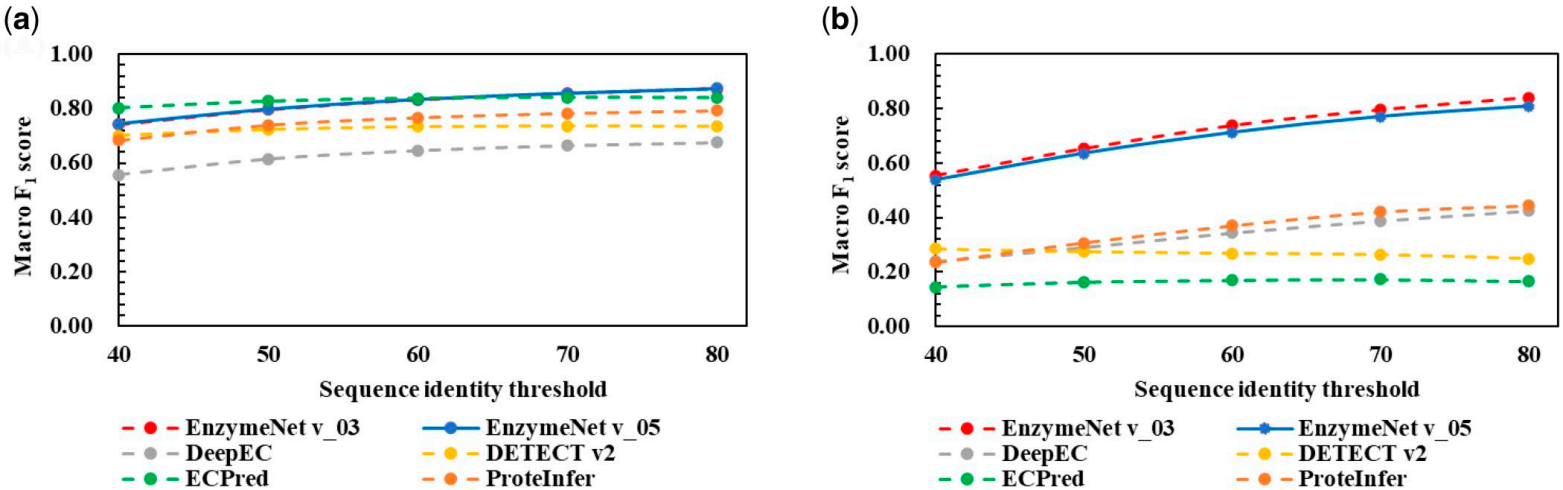
Macro *F*_1_ scores of (a) EC number first digit prediction and (b) complete EC number prediction using the common test and test sequences removing similar sequences to our training enzyme sequences for each sequence identity threshold using CD-HIT.

**Figure 6.**
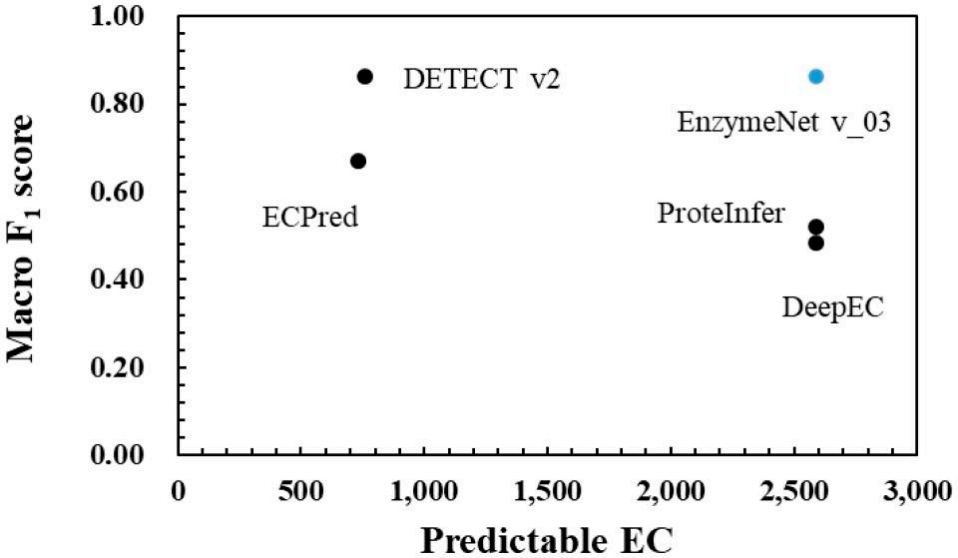
Macro *F*_1_ scores of the predictable EC numbers which each model outputted using the test data of complete EC number prediction. The number of predictable EC numbers in EnzymeNet models, DeepEC, DETECT v2, ECPred, and ProteInfer in fact is 2591, 4669, 764, 732, and 3411, respectively (Ryu *et al.* 2019; Sanderson et al. 2023).

Figure 5 shows the results of the two datasets, which similar sequences to the training datasets are removed from, in EC first digit and complete EC number predictions. The lower sequence identity threshold was, the more difficult the predictions were not depending on prediction models. In the first prediction, both EnzymeNet models predicted more correctly in 70 and 80 sequence identity thresholds. However, ECPred was the most accurate between all models. On the other hand, both EnzymeNet models were more accurate in the complete EC number prediction not depending on the value of sequence identity thresholds. All models in these evaluations showed the decreases in prediction accuracy as more similarity sequences were removed. Moreover, Fig. 6 shows the results of the predictable EC numbers outputted by each model using the test data of the second prediction to fairly compare the results. This is because the predictable EC numbers in the other models were not shown. DETECT v2 and EnzymeNet v_03 models are almost the same high accuracy although the number of predictable EC numbers in DETECT v2 was small. Finally, Supplementary Table S7 shows the Macro *F*_1_ score results of the EC numbers which could be more correctly predicted in EnzymeNet models and other models using *F*_1_ score of each EC number as a threshold. The previously reported models exhibited higher prediction accuracies in the limited EC number than EnzymeNet models.

## 4 Discussion

We present EnzymeNet models to predict complete EC number for each amino acid sequence in two-step prediction while removing non-enzyme proteins and exceptional sequences. To discover novel enzymes within a vast number of unannotated protein sequences, enzyme prediction models for enzyme functions need to efficiently learn the patterns of amino acids for each enzyme sequence. Therefore, EnzymeNet models were built to enable to remove the sequences with numerous consecutive identical amino acids, which are found within unannotated sequences, as well as non-enzyme proteins. The conventional EC number prediction models have not considered such sequences. Moreover, EnzymeNet models deeply learned various patterns of amino acid sequences by adding the random substitution sequences, which were similar to the original enzymes, to the datasets. To characterize more enzyme features, Positional Embedding layer which was included in relatively new models such as Transformer and Generative Pre-Training models was used in addition to ResNet structure (Vaswani *et al.* 2017, Radford *et al.* 2018). Therefore, EnzymeNet models learned position features of each amino acid of a protein which are important for protein activity, secondary structure, and protein–ligand interaction.

First, the methods of generating artificial negative sequences in the first prediction were optimized. All EnzymeNet models in the evaluations of test and common test data maintained high prediction accuracy. However, the prediction results of artificial negative sequences using common test data were significantly different depending on the models. EnzymeNet v_01 model which did not learn the artificial sequences did not predict almost the sequences. This is because machine learning models generally have difficulty predicting the data which is so different from training data.

Considering the results of the positive and negative data, two complete EC number prediction models were built based on EnzymeNet v_03 and v_05 models, which exhibited higher prediction accuracy of the overall sequences. Moreover, the artificial negative condition of EnzymeNet v_03 model is suitable for the prediction because the model predicted the consecutive substitution sequences constructed by all conditions with higher accuracy. The results of EnzymeNet v_03 models in the complete EC number prediction and the Continuous Test predictions were almost as accurate as those of EnzymeNet v_05 models. This indicates that the conditions of generating artificial negative samples do not have a significant influence on the overall prediction accuracy in both predictions. Moreover, the accuracies of EnzymeNet v_03 and v_05 models did not depend on the number of training data and the number of similar sequences (Supplementary Figs S6 and S7). These results show that the EnzymeNet models do not necessarily predict only easy EC numbers with high accuracy.

Next, the EnzymeNet models were compared with four previously reported EC number prediction models (Fig. 4a and Supplementary Fig. S5 and Table S6). In the prediction of common test data, EnzymeNet models exhibited higher prediction accuracy. Furthermore, the previously reported models could not predict the sequences with consecutive identical amino acids which are apparently non-enzyme. The results and common test results of EnzymeNet v_01 model indicate that prediction models cannot predict the exceptional sequences without learning them. However, the EnzymeNet models could not classify non-enzyme and random substitution sequences with the highest accuracy. Even though the number of non-enzyme sequences is clearly larger than that of enzyme sequences, EnzymeNet models learned more enzymes. The number of no-enzyme training data in ProteInfer was about 200 000 and was larger than that of our models. This is why the accuracies of the models for non-enzyme sequences were lower.

Moreover, EnzymeNet models had difficulty classifying the random substitution sequences because the models learned both random substitution sequences and the pre-substituted enzyme sequences (the enzyme sequences before substituting) which were similar to each other. Since the other models except for DeepEC were built from fewer enzyme sequences than EnzymeNet models, it is assumed that the other models did not learn the pre-substituted enzymes and were able to predict them without confusion. However, to improve the ability of our models to predict non-enzyme and random substitution sequences, the optimization of these datasets is required.

For complete EC number prediction, EnzymeNet models showed much higher prediction accuracy than the other models and the ensemble methods (Figs 4b and 6). Ensemble 1 using a majority rule of the four models was not improved, and Ensemble 2, combining the prediction ability of the models, improved prediction accuracy, yet the accuracy was lower than that of the EnzymeNet models. The results suggest that EnzymeNet models can predict the EC numbers which the other previously reported models have difficulty predicting. On the other hand, DeepEC and ProteInfer correctly classified positive enzymes because the Macro Precisions were much higher than Macro Recalls. Therefore, the putative enzymes which are predicted as positive by these models can be assigned new annotations. Moreover, the test enzyme sequences included in 271 EC number seem to be a core set that all models can predict with high accuracy according to Supplementary Table S7. For the prediction of this core set, DETECT v2 and the other reported models are superior to EnzymeNet models although EnzymeNet can predict extensive EC numbers combining the core set and the other enzyme sequences.

In addition, CLEAN (Yu et al. 2023), which has recently been developed, can predict single or multiple EC numbers for each sequence, although it cannot predict non-enzymes. Hence, EnzymeNet models were only compared to CLEAN in complete EC number prediction using the same test data. The test samples were regarded as correct in CLEAN if one of the multiple EC numbers output by CLEAN was successfully predicted. The test results are shown in Supplementary Fig. S8.

As a result, EnzymeNet models exhibited higher accuracy in complete EC prediction than CLEAN. However, the accuracy of CLEAN is higher in comparison to the other models. EnzymeNet models can predict a single enzyme function for each sequence. Therefore, the accuracy of CLEAN might have been lower because the prediction targets of CLEAN are a little different from that of EnzymeNet models. Considering the ability of CLEAN, which cannot predict non-enzyme, EnzymeNet firstly removes non-enzymes and exceptional sequences and predicts single enzyme functions when predicting enzyme functions for unannotated protein sequences. Next, CLEAN predicts detailed enzyme functions for the candidate enzyme sequences output from EnzymeNet model prediction. Therefore, candidate enzyme sequences for synthesis of target functional compounds can be selected using EnzymeNet and CLEAN.

The other EC number prediction models (Zou *et al.* 2019, Khan *et al.* 2021) were built from various enzyme features while EnzymeNet models, DeepEC, and ProteInfer need so simple features, namely, one-hot encoding, token, and positional embedding. These simple feature extractions do not have a large effect on prediction results, which depend on only amino acid pattern information. Moreover, the number of training data in DeepEC was almost as large as that of EnzymeNet models. This suggests that prediction accuracy does not necessarily rely on the number of training data for each model. Building optimized model structure to match prediction target is required.

Furthermore, EnzymeNet and the four models are evaluated using the datasets which similar sequences to the training datasets are removed from. EnzymeNet models also exhibited higher prediction accuracy for difficult enzyme sequences in EC number complete prediction even though the models did not show the highest accuracy in the first prediction. The results suggest EnzymeNet model v_03 model can more correctly predict EC numbers for more extensive sequences in comparison to reported models. However, EnzymeNet models cannot correctly predict some enzymes with lower similarity to training data and some EC numbers which DETECT v2 could predict with high accuracy. To improve the abilities of EnzymeNet models for the difficult positive, non-enzyme, and random substitution predictions further, updated methods to build training data and model structure are needed. The common decreases (Fig. 5) in prediction accuracy of all machine learning models except for DETECT v2 as lowering the threshold indicates that the evaluation of the difficult enzymes in the predictions may be insufficient.

In summary, EnzymeNet models can exclude the exceptional sequences from the sequence candidates in addition to the EC number prediction, which were more accurate for extensive enzyme sequences than the reported models. Moreover, up to 4000 sequences are predicted using our models in about 10 min at one time. Therefore, EnzymeNet models enable to apply to find available enzymes from metagenomics registered in sequences databases (Agarwala *et al.* 2018, Mitchell *et al.* 2020). In this case, to quickly predict EC numbers for a huge number of protein sequences, decreasing the training sequences included in some EC numbers whose number of sequences is larger is required. Moreover, for the putative enzyme sequences predicted using EnzymeNet models, the Substrate-Enzyme-Product models developed in our previous study (Watanabe *et al.* 2022) can predict corresponding substrates and products, namely, detailed enzymatic reaction annotations. The robustness of EnzymeNet models will lead to predict enzyme annotations related to enzymatic reactions for mass unannotated protein sequences and discover novel enzymes for biosynthesis of functional compounds using microorganisms.

## Supplementary Material

vbad173_Supplementary_Data

## Data Availability

The source codes of EnzymeNet models are freely available at https://github.com/nwatanbe/enzymenet.
