# Supplementary material for: EnzymeNet: residual neural networks model for Enzyme Commission number prediction
Source: Bioinform Adv. 2023 Nov 24;3(1):vbad173. doi: 10.1093/bioadv/vbad173 (PMC10701793; doi:10.1093/bioadv/vbad173)
Supplement: vbad173_Supplementary_Data [file vbad173_supplementary_data.zip › sup_EnzymeNet_3rd_manuscript_article_revision_1005.docx]

**EnzymeNet: Residual neural networks model for Enzyme Commission number prediction**

Naoki Watanabe, Masaki Yamamoto, Masahiro Murata, Yuki Kuriya, and Michihiro Araki^*^

**Supplementary Information**

**Performance evaluation parameters for test data**

To evaluate prediction model performance, the following values were calculated, given by:

$$Accuracy=\frac{The number of correct samples}{The number of test samples}$$

$$Precision=\frac{TP}{TP+FP}$$

$$Recall=\frac{TP}{TP+FN}$$

$$F_{1} score=\frac{2\cdot Precision\cdot Recall}{Precision+Recall}$$

$$MCC=\frac{TP\cdot TN-FP\cdot FN}{\sqrt{\left( TP+FP \right)\cdot\left( TP+FN \right)\cdot\left( TN+FP \right)\cdot\left( TN+FN \right))}}$$

where $TP$, *TN*, $FP$, and $FN$ represent true positives, true negatives, false positives and false negatives, respectively. TP and TN were the number of samples that are correctly predicted, while FP and FN were the number of samples that are incorrectly predicted. The values below were also calculated as given by:

$$Macro Precision=\frac{1}{L}\sum_{i=1}^{L} {Precision}_{i}$$

$$Macro Recall=\frac{1}{L}\sum_{i=1}^{L} {Recall}_{i}$$

$$Macro F_{1} score=\frac{2\cdot Macro Precision\cdot Macro Recall}{Macro Precision+Macro Recall}$$

The values were calculated using the scikit-learn library(Pedregosa *et al.*, 2011).

**Supplementary Figures**

**Supplementary Figure S1.** Pie chart of registration status of the EnzymeNet data (Table 1) in (A) National Center for Biotechnology Information (NCBI) and (B) UniProt.


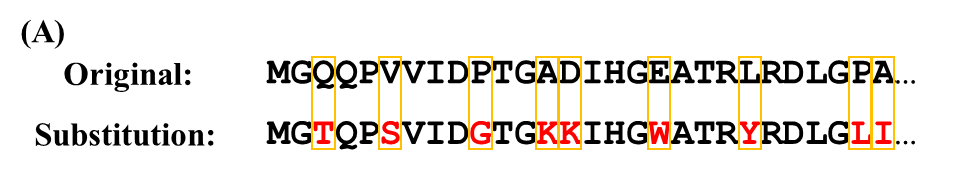


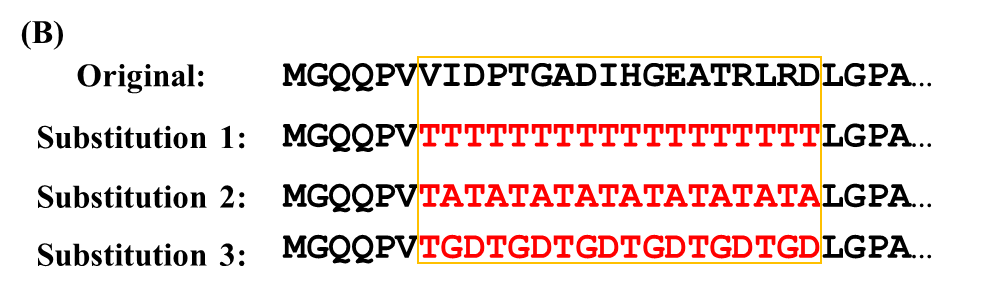


**Supplementary Figure S2.** Examples of (A) Random substitution and (B) Consecutive substitution. The substituted positions of amino acids are surrounded by orange-bordered squares and substituted amino acids are marked in red color.


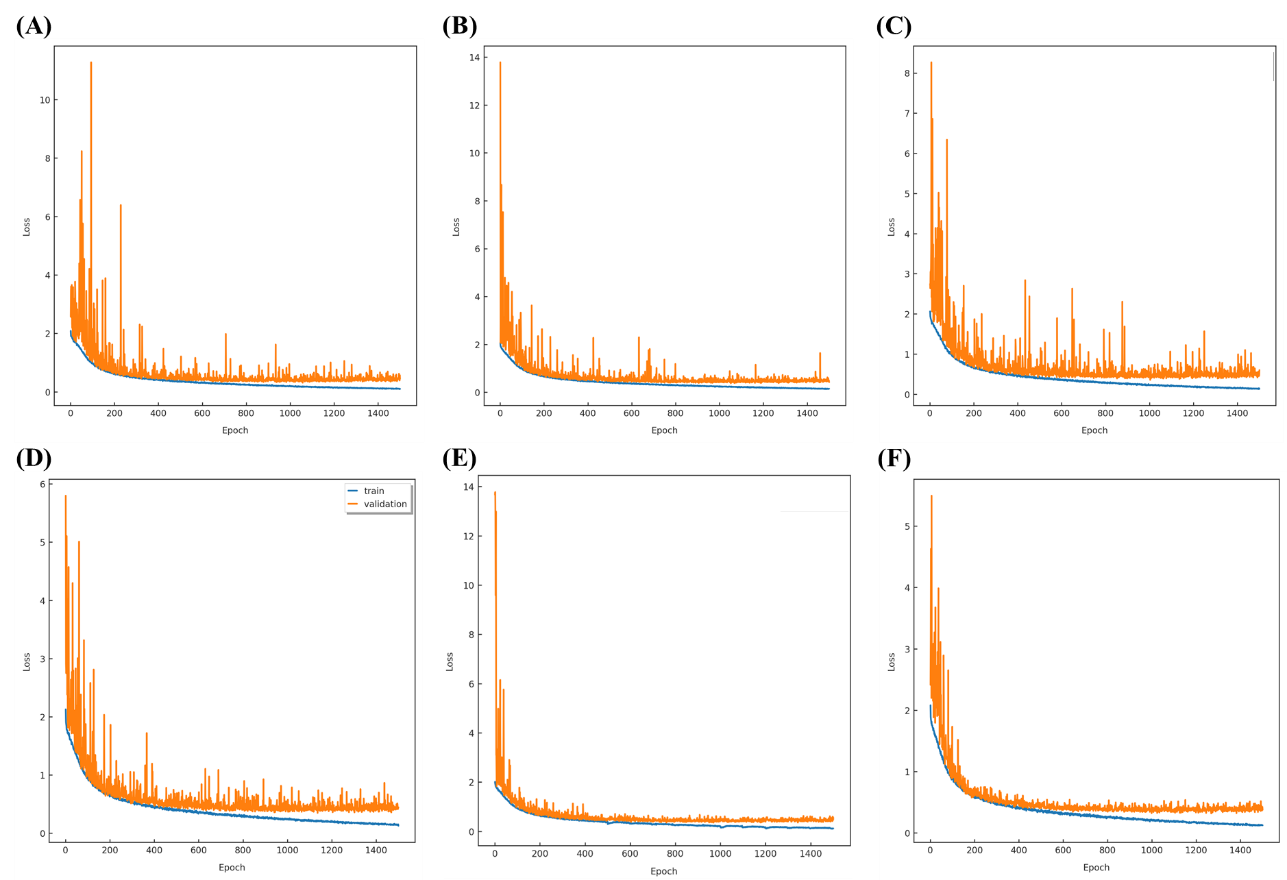


**Supplementary Figure S3.** Training and validation loss curves (blue line: training, orange line: validation) of the EnzymeNet models in the first prediction using 6 different negative datasets for 1,500 epochs. (A) EnzymeNet version_01 (v_01) model, (B) EnzymeNet v_02 model, (C) EnzymeNet v_03 model, (D EnzymeNet v_04 model, (E) EnzymeNet v_05 model, (F) EnzymeNet v_06 model as shown in Table 2.


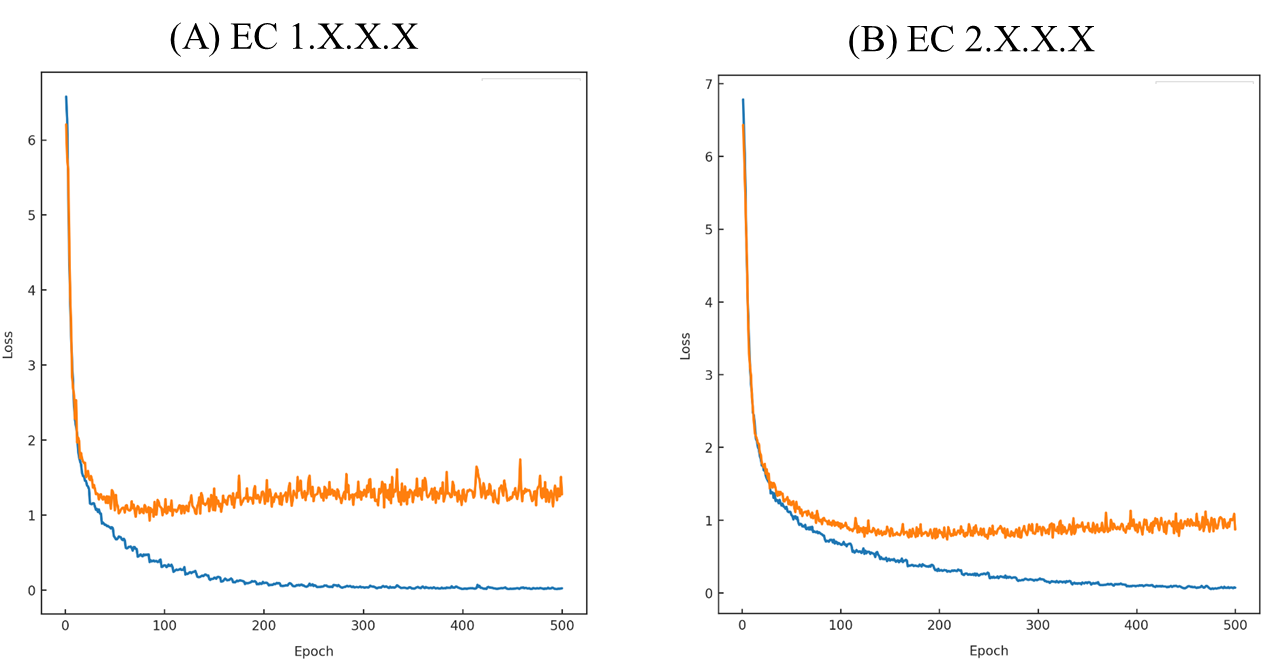

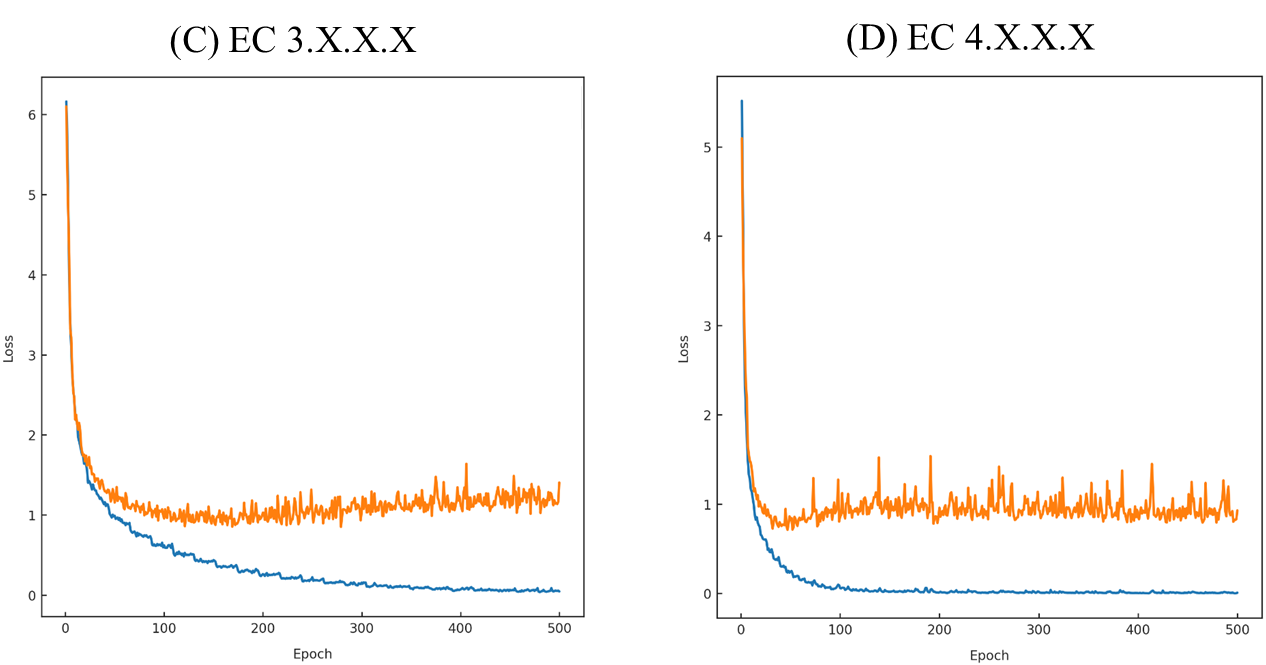

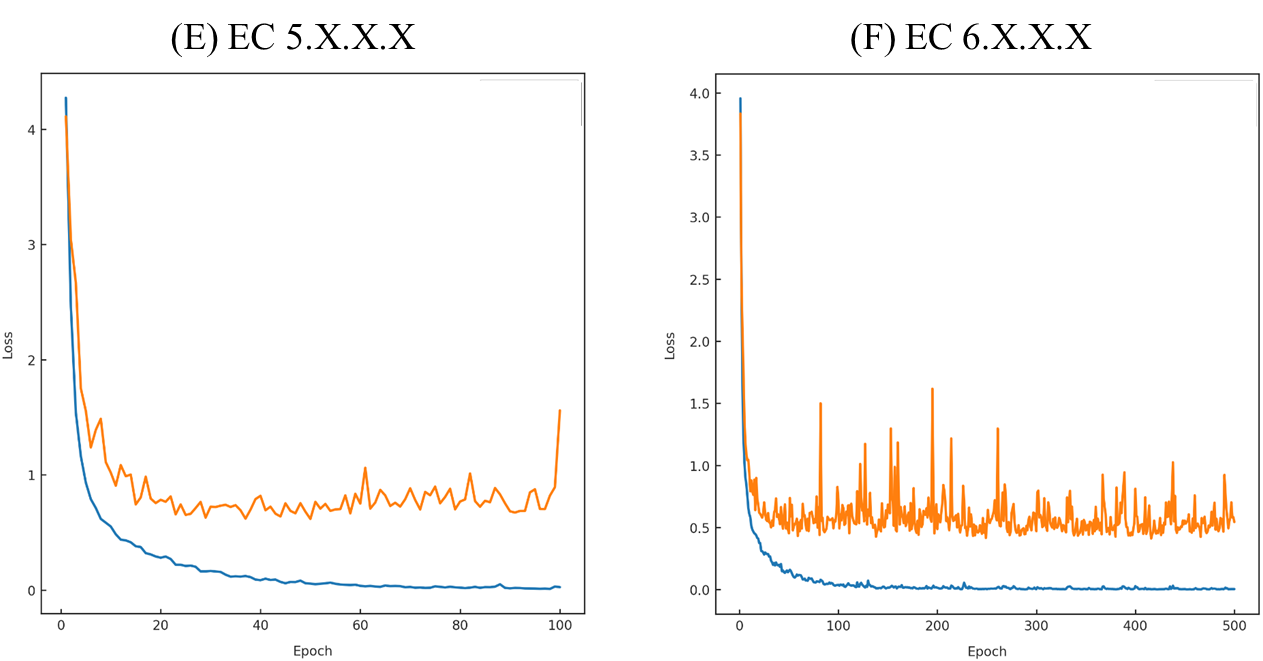


**Supplementary Figure S4.** Training and validation loss curves (blue line: training, orange line: validation) of each model in the second prediction using EnzymeNet v_05 model. (A) EC 1, (B) EC 2, (C) EC 3, (D) EC 4, (E) EC 5 and (F) EC 6.

**Supplementary Figure S5.** Comparative common test results of correct prediction rate in only negative prediction. The number of non-enzyme test sequences was 14,272 and the number of random substitution test sequences and consecutive substitution test sequences were 1,674, respectively.

**Supplementary Figure S6.** Relationship between EnzymeNet’s accuracies in complete EC number prediction and the number of training data for each EC number. Spearman’s rank correlation coefficients were used. (A) The accuracies of EnzymeNet v_03 model, (B) The accuracies of EnzymeNet v_05 model.

**Supplementary Figure S7.** Relationship between EnzymeNet’s accuracies in complete EC number prediction and the values of sequence similarity rate for each EC number. Spearman’s rank correlation coefficients were used. (A) The accuracies of EnzymeNet v_03 model, (B) The accuracies of EnzymeNet v_05 model. Each similarity rate was calculated from the results of 80 % identity using CD-HIT(Li and Godzik, 2006) for each EC number and is defined as follows:

$$Similarity rate=1-\frac{The sum of the enzyme sequences that did not get clustered}{The sum of enzyme sequences}$$

$$(For each EC number)$$

where the sum of sequences means the number of enzyme sequences included in each EC number. The clusters were built from enzyme sequences included in each EC number by CD-HIT, and then the number of the cluster included in only 1 enzyme sequence for each EC number was counted.

**Supplementary Figure S8.** Comparative results of complete EC number prediction between EnzymeNet models and CLEAN (Yu *et al.*, 2023).

**Supplementary Tables**

**Supplementary Table S1. Common test data size.**

|  | Common test |
| --- | --- |
| EC1 | 22,161 |
| EC2 | 34,473 |
| EC3 | 25,418 |
| EC4 | 10,054 |
| EC5 | 6,182 |
| EC6 | 5,489 |
| Non-enzyme | 14,272 |
| Random substitution 10% | 558 |
| Random substitution 20% | 558 |
| Random substitution 40% | 558 |
| Consecutive substitution 1~25% | 558 |
| Consecutive substitution 26~49% | 558 |
| Consecutive substitution 50~80% | 558 |

**Supplementary Table S2. Test results of EC number first digit prediction using 6 EnzymeNet models for 1,500 epochs.**

| EnzymeNet v_01 model | | | | | EnzymeNet v_02 model | | | | |
| --- | --- | --- | --- | --- | --- | --- | --- | --- | --- |
| Epoch | Macro F_1_ score | Macro Precision | Macro Recall | MCC | Epoch | Macro F_1_ score | Macro Precision | Macro Recall | MCC |
| 100 | 0.601 | 0.571 | 0.634 | 0.494 | 100 | 0.596 | 0.639 | 0.558 | 0.438 |
| 200 | 0.776 | 0.779 | 0.773 | 0.691 | 200 | 0.733 | 0.735 | 0.732 | 0.641 |
| 300 | 0.735 | 0.714 | 0.758 | 0.656 | 300 | 0.761 | 0.731 | 0.794 | 0.743 |
| 400 | 0.802 | 0.783 | 0.822 | 0.751 | 400 | 0.843 | 0.847 | 0.839 | 0.768 |
| 500 | 0.817 | 0.792 | 0.843 | 0.765 | 500 | 0.841 | 0.840 | 0.841 | 0.771 |
| 600 | 0.860 | 0.856 | 0.865 | 0.815 | 600 | 0.841 | 0.837 | 0.845 | 0.800 |
| 700 | 0.860 | 0.841 | 0.881 | 0.830 | 700 | 0.853 | 0.855 | 0.852 | 0.788 |
| 800 | 0.874 | 0.871 | 0.876 | 0.838 | 800 | 0.841 | 0.834 | 0.849 | 0.827 |
| 900 | 0.882 | 0.884 | 0.879 | 0.846 | 900 | 0.857 | 0.853 | 0.861 | 0.822 |
| 1,000 | 0.886 | 0.883 | 0.888 | 0.850 | 1,000 | 0.868 | 0.876 | 0.860 | 0.807 |
| 1,100 | 0.887 | 0.892 | 0.882 | 0.849 | 1,100 | 0.875 | 0.880 | 0.870 | 0.805 |
| 1,200 | 0.886 | 0.879 | 0.894 | 0.853 | 1,200 | 0.869 | 0.867 | 0.870 | 0.829 |
| 1,300 | 0.857 | 0.832 | 0.884 | 0.834 | 1,300 | 0.878 | 0.872 | 0.884 | 0.834 |
| 1,400 | 0.889 | 0.881 | 0.897 | 0.862 | 1,400 | 0.860 | 0.854 | 0.866 | 0.833 |
| 1,500 | 0.898 | 0.897 | 0.899 | 0.868 | 1,500 | 0.895 | 0.903 | 0.888 | 0.842 |

| EnzymeNet v_03 model | | | | | EnzymeNet v_04 model | | | | |
| --- | --- | --- | --- | --- | --- | --- | --- | --- | --- |
| Epoch | Macro F_1_ score | Macro Precision | Macro Recall | MCC | Epoch | Macro F_1_ score | Macro Precision | Macro Recall | MCC |
| 100 | 0.597 | 0.626 | 0.571 | 0.448 | 100 | 0.612 | 0.638 | 0.587 | 0.470 |
| 200 | 0.736 | 0.733 | 0.739 | 0.653 | 200 | 0.729 | 0.700 | 0.761 | 0.660 |
| 300 | 0.800 | 0.799 | 0.801 | 0.746 | 300 | 0.765 | 0.738 | 0.794 | 0.706 |
| 400 | 0.788 | 0.764 | 0.814 | 0.732 | 400 | 0.769 | 0.737 | 0.804 | 0.710 |
| 500 | 0.825 | 0.825 | 0.825 | 0.777 | 500 | 0.790 | 0.759 | 0.823 | 0.737 |
| 600 | 0.828 | 0.803 | 0.854 | 0.785 | 600 | 0.820 | 0.798 | 0.843 | 0.777 |
| 700 | 0.860 | 0.867 | 0.852 | 0.808 | 700 | 0.842 | 0.825 | 0.860 | 0.796 |
| 800 | 0.864 | 0.864 | 0.864 | 0.818 | 800 | 0.807 | 0.785 | 0.831 | 0.757 |
| 900 | 0.863 | 0.867 | 0.860 | 0.813 | 900 | 0.841 | 0.818 | 0.865 | 0.802 |
| 1000 | 0.872 | 0.877 | 0.868 | 0.829 | 1000 | 0.860 | 0.848 | 0.872 | 0.825 |
| 1100 | 0.814 | 0.798 | 0.831 | 0.763 | 1100 | 0.827 | 0.797 | 0.859 | 0.797 |
| 1200 | 0.881 | 0.887 | 0.874 | 0.845 | 1200 | 0.869 | 0.861 | 0.877 | 0.834 |
| 1300 | 0.859 | 0.841 | 0.877 | 0.831 | 1300 | 0.830 | 0.815 | 0.845 | 0.781 |
| 1400 | 0.881 | 0.888 | 0.874 | 0.848 | 1400 | 0.863 | 0.849 | 0.877 | 0.828 |
| 1500 | 0.852 | 0.846 | 0.858 | 0.812 | 1500 | 0.870 | 0.857 | 0.883 | 0.843 |

| EnzymeNet v_05 model | | | | | EnzymeNet v_06 model | | | | |
| --- | --- | --- | --- | --- | --- | --- | --- | --- | --- |
| Epoch | Macro F_1_ score | Macro Precision | Macro Recall | MCC | Epoch | Macro F_1_ score | Macro Precision | Macro Recall | MCC |
| 100 | 0.645 | 0.652 | 0.638 | 0.511 | 100 | 0.645 | 0.652 | 0.638 | 0.511 |
| 200 | 0.754 | 0.75 | 0.759 | 0.662 | 200 | 0.754 | 0.75 | 0.759 | 0.662 |
| 300 | 0.771 | 0.75 | 0.793 | 0.699 | 300 | 0.771 | 0.75 | 0.793 | 0.699 |
| 400 | 0.810 | 0.793 | 0.828 | 0.761 | 400 | 0.810 | 0.793 | 0.828 | 0.761 |
| 500 | 0.833 | 0.823 | 0.844 | 0.793 | 500 | 0.833 | 0.823 | 0.844 | 0.793 |
| 600 | 0.792 | 0.773 | 0.813 | 0.734 | 600 | 0.792 | 0.773 | 0.813 | 0.734 |
| 700 | 0.857 | 0.87 | 0.844 | 0.795 | 700 | 0.857 | 0.87 | 0.844 | 0.795 |
| 800 | 0.872 | 0.881 | 0.864 | 0.835 | 800 | 0.872 | 0.881 | 0.864 | 0.835 |
| 900 | 0.869 | 0.872 | 0.866 | 0.818 | 900 | 0.869 | 0.872 | 0.866 | 0.818 |
| 1000 | 0.875 | 0.888 | 0.862 | 0.827 | 1000 | 0.875 | 0.888 | 0.862 | 0.827 |
| 1100 | 0.867 | 0.878 | 0.856 | 0.813 | 1100 | 0.867 | 0.878 | 0.856 | 0.813 |
| 1200 | 0.878 | 0.871 | 0.886 | 0.847 | 1200 | 0.878 | 0.871 | 0.886 | 0.847 |
| 1300 | 0.850 | 0.82 | 0.883 | 0.83 | 1300 | 0.850 | 0.82 | 0.883 | 0.83 |
| 1400 | 0.885 | 0.888 | 0.883 | 0.854 | 1400 | 0.885 | 0.888 | 0.883 | 0.854 |
| 1500 | 0.885 | 0.889 | 0.881 | 0.849 | 1500 | 0.885 | 0.889 | 0.881 | 0.849 |

**Supplementary Table S3. Common test results of prediction of artificial negative data.**

| Model | Random 10% | Random 20% | Random 40% | Consecutive 1 ~ 25% | Consecutive 26 ~ 49% | Consecutive 50 ~ 80% |
| --- | --- | --- | --- | --- | --- | --- |
| EnzymeNet v_01 | 0.072 | 0.095 | 0.102 | 0.215 | 0.606 | 0.862 |
| EnzymeNet v_02 | 0.188 | 0.376 | 0.765 | 0.527 | 0.964 | 0.995 |
| EnzymeNet v_03 | 0.197 | 0.380 | 0.758 | 0.760 | 0.973 | 0.998 |
| EnzymeNet v_04 | 0.109 | 0.229 | 0.412 | 0.351 | 0.891 | 0.995 |
| EnzymeNet v_05 | 0.136 | 0.324 | 0.688 | 0.428 | 0.910 | 0.993 |
| EnzymeNet v_06 | 0.111 | 0.297 | 0.785 | 0.430 | 0.925 | 0.996 |

Random: Random substitution, Consecutive: Consecutive substitution.

**Supplementary Table S4. Common test results of the first prediction for each class using optimized 2 EnzymeNet models.**

| EnzymeNet v_03 model | | | | | EnzymeNet v_05 model | | | | |
| --- | --- | --- | --- | --- | --- | --- | --- | --- | --- |
| Class | F_1_ score | Precision | Recall | Class | | F_1_ score | Precision | Recall |  |
| EC1 | 0.909 | 0.892 | 0.928 | EC1 | | 0.917 | 0.951 | 0.885 |  |
| EC2 | 0.898 | 0.909 | 0.887 | EC2 | | 0.890 | 0.861 | 0.921 |  |
| EC3 | 0.881 | 0.886 | 0.876 | EC3 | | 0.881 | 0.870 | 0.893 |  |
| EC4 | 0.904 | 0.904 | 0.903 | EC4 | | 0.900 | 0.950 | 0.855 |  |
| EC5 | 0.924 | 0.929 | 0.919 | EC5 | | 0.913 | 0.900 | 0.926 |  |
| EC6 | 0.890 | 0.963 | 0.828 | EC6 | | 0.894 | 0.860 | 0.929 | |
| Negative | 0.780 | 0.758 | 0.804 | Negative | | 0.780 | 0.807 | 0.754 | |

**Supplementary Table S5. Test Results of complete EC number prediction. (A) EnzymeNet v_03 model and (B) EnzymeNet v_05 model.**

| (A) EnzymeNet v_03 model | | | | | |
| --- | --- | --- | --- | --- | --- |
| Class | Epoch | Macro F_1_ score | Macro Precision | Macro Recall | MCC |
| EC1 | 500 | 0.860 | 0.872 | 0.849 | 0.837 |
| EC2 | 500 | 0.873 | 0.884 | 0.863 | 0.861 |
| EC3 | 400 | 0.852 | 0.857 | 0.847 | 0.838 |
| EC4 | 350 | 0.891 | 0.900 | 0.881 | 0.885 |
| EC5 | 90 | 0.927 | 0.934 | 0.921 | 0.925 |
| EC6 | 450 | 0.954 | 0.961 | 0.947 | 0.955 |

| (B) EnzymeNet v_05 model | | | | | |
| --- | --- | --- | --- | --- | --- |
| Class | Epoch | Macro F_1_ score | Macro Precision | Macro Recall | MCC |
| EC1 | 400 | 0.842 | 0.858 | 0.827 | 0.820 |
| EC2 | 500 | 0.865 | 0.872 | 0.858 | 0.852 |
| EC3 | 400 | 0.838 | 0.852 | 0.825 | 0.818 |
| EC4 | 400 | 0.880 | 0.889 | 0.871 | 0.869 |
| EC5 | 90 | 0.897 | 0.909 | 0.886 | 0.887 |
| EC6 | 300 | 0.928 | 0.934 | 0.923 | 0.933 |

**Supplementary Table S6. Comparative common test results of prediction of artificial negative data.**

| Model | Random 10% | Random 20% | Random 40% | Consecutive 1~25% | Consecutive 26~49% | Consecutive 50~80% |
| --- | --- | --- | --- | --- | --- | --- |
| EnzymeNet v_03 | 0.197 | 0.380 | 0.758 | 0.760 | 0.973 | 0.998 |
| EnzymeNet v_05 | 0.136 | 0.324 | 0.688 | 0.428 | 0.910 | 0.993 |
| DeepEC | 0.165 | 0.152 | 0.167 | 0.199 | 0.294 | 0.638 |
| DETECT v2 | 0.532 | 0.572 | 0.731 | 0.514 | 0.554 | 0.683 |
| ECPred | 0.050 | 0.054 | 0.097 | 0.100 | 0.194 | 0.425 |
| ProteInfer | 0.348 | 0.545 | 0.826 | 0.444 | 0.728 | 0.751 |

**Supplementary Table S7. Macro F_1_ score test results of the EC numbers which could be more correctly predicted by all models by using F_1_ score of each EC number as a threshold.**

| Threshold of Macro F_1_ score | Number of EC numbers | Macro F_1_ score | | | | | |
| --- | --- | --- | --- | --- | --- | --- | --- |
|  |  | EnzymeNet v_03 | EnzymeNet v_05 | DeepEC | DETECT v2 | ECPred | Proteinfer |
| 0.9 | 72 | 0.959 | 0.961 | 0.951 | 0.992 | 0.961 | 0.968 |
| 0.8 | 184 | 0.934 | 0.932 | 0.919 | 0.977 | 0.932 | 0.942 |
| 0.7 | 271 | 0.918 | 0.914 | 0.886 | 0.963 | 0.904 | 0.917 |
| 0.6 | 343 | 0.905 | 0.901 | 0.856 | 0.949 | 0.879 | 0.894 |

**Reference**

Li,W. and Godzik,A. (2006) Cd-hit: A fast program for clustering and comparing large sets of protein or nucleotide sequences. *Bioinformatics*, **22**, 1658–1659.

Pedregosa,F. *et al.* (2011) Scikit-learn: Machine learning in Python. *J. Mach. Learn. Res.*, **12**, 2825–2830.

Yu,T. *et al.* (2023) Enzyme function prediction using contrastive learning. *Science*, **379**, 1358–1363.
